# Correlation between depressive symptoms and subjective mastication ability and ability to pronunciation among Korean elderly

**DOI:** 10.4178/epih.e2016035

**Published:** 2016-07-25

**Authors:** Min Sun Park, Kyung-Gyun Hwang, Bo Youl Choi

**Affiliations:** 1 Institute for Health and Society, Hanyang University, Seoul, Korea; 2 Department of Dentistry/Oral and Maxillofacial Surgery, Hanyang University College of Medicine, Seoul, Korea; 3 Department of Preventive Medicine, Hanyang University College of Medicine, Seoul, Korea

**Keywords:** Elderly, Oral health, Chewing difficulty, Pronunciation discomfort, Depressive symptoms

## Abstract

**OBJECTIVES:**

The present study examines the relationship between depressive symptoms and subjective chewing and pronunciation ability in Korean seniors. Our goal is to provide the data required to develop appropriate oral health interventions programs for seniors.

**METHODS:**

The Center for Epidemiologic Studies-Depression Scale (CES-D) is widely used depressive symptoms assessment. A Korean version was used for the 2009 Community Health Survey, which was consulted to extract the present study’s participants comprising 50,694 Korean seniors (males, 20,582; females, 30,112) aged ≥65 years. Those with a CES-D score ≥16 were rated ‘depressed.’ SAS version 9.3 was used for the data analysis.

**RESULTS:**

Prevalence of depressive symptoms increased as the participants socioeconomic status decreased, number of health issues increased, health behavior worsened, and chewing and pronunciation discomfort increased. Males with chewing difficulties were found to have 1.45 times (95% confidence interval [CI], 1.29 to 1.63) greater risk of depressive symptoms than those without, while males with pronunciation discomfort were found to have 1.97 times greater risk of depressive symptoms than those without (95% CI, 1.76 to 2.20). Females with chewing difficulty were found to have 1.50 times (95% CI, 1.39 to 1.61) greater risk of depressive symptoms than those without, and females with pronunciation discomfort were found to have 1.55 times greater risk of depressive symptoms than those without (95% CI, 1.44 to 1.67).

**CONCLUSIONS:**

Intervention programs designed to help with oral health management and alleviate depressive symptoms in seniors are urgently needed. As the prevalence of depressive symptoms may vary geographically, research examining potential variance at city, district, and town levels would be beneficial.

## INTRODUCTION

Statistics Korea data (2011) describe decreasing birth rate and increasing life expectancy as reshaping the nation’s population pyramid into one which is narrower at the base and wider at the top (old-age) [1]. Elderly persons exhibit characteristics including function impairments/loss, bedridden lifestyles, long-term dietary and nutritional issues, and incurable chronic conditions [2]. The National Insurance Review and Assessment Service (2014) suggest individuals aged 65 years and older account for 11.95% of the nation’s population−a 0.4% increase from the previous year−and were responsible for 35.5% of total healthcare cost, a 1.0% increase from the previous year. Chronic gingivitis and other periodontal diseases increased the outpatient healthcare cost to 122.3 billion Korean won (KRW) annually (30.1% increase), with a per-patient healthcare cost of 68,000 KRW [3].

Oral health is one of the most fundamental measures of a person’s overall health: unhealthy teeth interfere with nutritional intake, subsequently undermining good health. Because the mouth and teeth play a critical role in chewing, tasting, and pronunciation, we cannot discuss health without mentioning oral health. The Korea Centers for Disease Control and Prevention (2015) suggest up to half of Korean elderly persons aged ≥65 years have less than 20 natural teeth. Despite increasing efforts to restore teeth function, evidenced by growing rates of dental implants, approximately half of the elderly population experience difficulty chewing [4]. Tooth loss diminishes one’s mastication ability and restricts the range of food choices, subsequently lowering the quantity/quality of nutritional intake, making it difficult to maintain good health [5]. As the elderly population grew, so did the number of patients complaining of dry mouth and related symptoms. Chronic reduced salivary flow can cause discomfort when articulating and chewing/swallowing food; it can also irritate and inflame the mouth’s soft tissues due to dryness. Without sufficient saliva flow, the mouth is less able to clean and shield itself from tooth decay, gum disease, and bad breath, leaving it more susceptible to infections such as oral candidiasis [6]. As such, continued efforts are needed to improve the oral health of elderly persons.

Depression is feelings of sadness and despair that impair normal daily life lasting for two weeks or more. National Health and Nutrition Examination Survey (2012) state rates of depression are 1.8 times greater among Korean males than Korean females. The rate increased with age, peaking among males and females aged ≥70 years [7].

Examining the correlation between depression and ability to mastication and pronunciation for managing depression associated with oral health in elderly persons. In some areas of Korea, the Oral Health Impact Profile-14 of elderly persons attending senior citizen centers is found to be closely associated with depression, measured by the Geriatric Depression Scale-Short Form and Quality of Life scales [8]. It is also reported that oralexercise programs for the elderly (held in community health centers around the nation) improved Short-form Geriatric Depression Scale scores for elderly people [9]. However, the research had a limited regional focus, making it difficult to generalize the findings. Depression is also reported to be significantly associated with oral health status and behavior (e.g., subjective health status, chewing difficulty, tooth pain, and temporomandibular joint dysfunction). However, the study failed to reflect the lifecycle characteristics of elderly people due to grouping them with the general adult population (aged ≥19 years). Furthermore, the study participants depression status was determined through a single questionnaire item inquiring whether they had ever experienced depressive symptoms in his/her lifetime thus far. As for international research, oral health status (Geriatric Oral Health Assessment Index) has been associated with depression among elderly German people (Self-rating Depression Scale) [10], and saliva flow, oral pain, and chewing difficulty have been associated with depression in elderly Japanese people [11]. However, potential differences in culture and lifecycle characteristics make it hard to generalize these findings to the elderly Korean population. There are also recent Korean studies reporting on the impact of oral health on depression among the elderly; however, their findings are also insufficient for generalization as the studies’ scope were geographically limited. The present study examines correlations between depressive symptoms and subjective mastication ability and ability to pronounce in the elderly Korean population. Its aim is to provide foundational data required to develop depressive symptoms and oral health management intervention programs for the elderly. Participants were stratified by sex to investigate potential sex differences in depressive symptoms status and ability to chew and pronounce.

## MATERIALS AND METHODS

### Study participants and survey method

Data was collected using paper-assisted personal interviewing as part of the Korea Centers for Disease Control and Prevention’s 2009 Community Health Survey (CHS). A total of 253 Korean community health centers and 35 Korean universities participated in the survey conducted between September and November 2009.

Participants consisted of Korean adults aged ≥19 years. Trained personnel conducted personal interviews at each household within the sample. On average, 900 participants per health center are surveyed each year. Uninhabitable redevelopment/reconstruction areas, sparsely populated commercial and manufacturing areas, Hansen villages, dormitories, and religious communes were all excluded from the sample. Due to the nature of one-on-one interviewing, the following were also excluded from the sample: those not available for three or more contacts, those expecting to be out of town or overseas for a prolonged period of time, and those not available during the survey period due to medical reasons (CHS raw data use guidelines, 2009).

The widely used Center for Epidemiologic Studies-Depression Scale (CES-D) was translated into Korean for the 2009 CHS. In total, 50,694 2009 CHS respondents (20,582 males; 30,112 females) aged ≥65 years were selected as participants. They were divided into two age groups: a ‘younger’ group of those aged 65 to 74 years (born between July 31,1934 and July 31, 1944) and an ‘older’ group of those aged ≥75 years (born before July 31, 1934).

### Ethics statement

The study protocol was approved by the institutional review board (IRB) of Hanyang University (IRB no. HYI-15-215-1).

### Survey tool

#### Subjective chewing and pronunciation ability

The 2009 CHS asked respondents, “Are you currently experiencing chewing difficulty or discomfort due to problems relating to your teeth, dentures, or gums?” and “Are you currently experiencing difficulty or discomfort with clear pronunciation due to problems relating to your teeth, dentures, or gums?” For both questions, respondents were given the following instruction: “If wearing dentures, please answer the above question regardless.” Response choices included “not at all”,“ moderately so”, “a little bit”, which were coded as “no”, and “I experience difficulty” and “very much so”, which were coded as “yes.”

#### Depressive symptoms

Depressive symptoms were measured with the CES-D developed by Radloff [12] translated into Korean by Cho & Kim [13]. The CES-D is a self-reporting depression screening tool comprising 20 items. Each item is scored out of three, ranging from ‘never’ and ‘almost never: less than once per week’ (0 point), ‘a little bit: 1-2 times per week’ (1 point), ‘occasionally/frequently: 3-4 times a week’ (2 points), and ‘most of the time: more than 5 times a week’ (3 points). The total score range is 0-60 points, with higher scores indicating greater levels of depressive symptoms.

#### Demographic variables, disease morbidity, health level, and health behavior

Participants were stratified by sex and age. Individuals born between July 31, 1974 and July 31, 1944 (65-74-years-old) were grouped in the ‘younger’ bracket, and those born before July 31, 1934 (75+-years-old) grouped in the ‘older’ bracket. Education levels were divided into ‘no education’, ‘elementary to middle school education’, ‘high school education’, or ‘more’. Participants National Basic Livelihood Security (NBLS) benefit recipient status was assessed with either ‘yes’ or ‘no.’

Chronic disease accounted for were hypertension, diabetes, high cholesterol, stroke, cardiomyopathy, angina, arthritis, osteoporosis, tuberculosis, asthma, and rhinitis. Respondents scored either ‘0’, ‘1-2’, or ‘3 or more’ relating to the number of the above diagnoses they had. For the item relating to the level of stress perceived throughout daily life the answers ‘almost never’ or ‘a little bit’ were coded as ‘no’, and the answers ‘tend to feel a lot (of stress)’ and ‘a great deal’ were coded as ‘yes.’

For the item relating to smoking status, those who answered ‘does not apply’ were grouped into ‘non-smokers’, those who answered ‘smoked in the past, but not currently’ were grouped as ‘previous smokers’, and those who answered ‘smoking daily’ and ‘smoking occasionally’ were grouped as ‘current smokers.’ For the item pertaining to alcohol use during the previous year, participants who answered ‘not applicable’ were classified as ‘non-drinkers,’ those who answered ‘yes’ were classified as ‘current drinkers’, and those who answered ‘no’ were classified as ‘previous drinkers.’ Sleeping hours referred to the average number of hours spent sleeping, which excluded napping during the daytime hours. This was classified into ‘less than 6 hours’, ‘6-8 hours’, and ‘8 hours and more’.

### Data analysis

Descriptive analysis was performed to examine participants general characteristics, and chi-square analysis to examine the factors influencing chewing and pronunciation discomforts and rate of depressive symptoms. Multiple regression analysis was conducted to identify correlations between oral health and depressive symptoms, with 95% confidence intervals (CIs) calculated controlling for demographic factors, disease morbidity, health level, and health behavior. The analysis was sex stratified, and sleeping hours were analyzed as quartiles. Statistical analysis was performed using SAS version 9.3 (SAS Institute Inc., Cary, NC, USA) .

## RESULTS

### Participants general characteristics

The sample’s proportion of females was higher than that of males in participants ≥65 years old. Regarding males, 48.9% complained of chewing difficulty, 25.6% of pronunciation discomfort, and 12.7% reported depressive symptoms (CES-D 16 points or more). The proportion of ‘younger’ males was greater than the ‘older’ males. Regarding demographics, 73.3% reported having middle school education or less, and 6.5% reported receiving NBLS benefits. Over half reported at least one chronic condition, 10.8% reported three or more, and the majority reported a relatively low level of stress. The proportion who were smokers was greater than non-smokers; and among the smokers, the number of previous smokers was high. As for alcohol use, the proportion of current drinkers was the highest. Those suspected to be either sleep-deprived or sleeping too much accounted for 27.6% (Table 1).

Regarding females, more complained of chewing difficulty (55.3%) than pronunciation discomfort (28.2%), and 23.3% reported depressive symptoms. Similar to male participants, the proportion of ‘younger’ females were greater than the ‘older’ females. Regarding demographics, 94.8% reported middle school education or less, and 11.4% reported receiving NBLS benefits. Over half reported one or more chronic conditions, 25.5% reported three or more, and the majority reported relatively low levels of stress. The proportion of non-smokers was greater than those who have smoked. Those suspected to be sleep-deprived or to be sleeping too much accounted for 32.9% (Table 1).

### Key factors influencing chewing difficulty

Age-adjusted prevalence of chewing difficulty increased as education level decreased, and was higher among recipients of NBLS benefits. Chewing difficulty increased as the number of chronic diseases and levels of stress increased. Male smokers reported greater levels of chewing difficulty than those who smoked in the past. Previous drinkers, and those who are sleep-deprived or sleeping too much, all reported high levels of chewing difficulty. Similar patterns were seen across both sexes (Table 2).

### Key factors influencing pronunciation discomfort

Age-adjusted pronunciation discomfort increased in both sexes, as education level decreased, and was higher among recipients of NBLS benefits. Pronunciation discomfort increased as number of chronic diseases and levels of stress increased. Greater levels of pronunciation discomfort were reported by current male and female smokers. Previous drinkers reported greater levels of pronunciation discomfort. Participants who were sleep-deprived or sleeping too much also reported greater levels of pronunciation discomfort, with similar patterns seen across both sexes (Table 3).

### Key factors influencing depressive symptoms

Age-adjusted prevalence of depressive symptoms increased in both sexes, as education level decreased, and was higher among recipients of NBLS benefits. depressive symptoms increased as level of stress increased. Current smokers and previous drinkers reported greater levels of depressive symptoms than those who had used tobacco in the past, and those who were current drinkers. Similar patterns were seen in both sexes. depressive symptoms was higher among participants who were sleep-deprived, sleep too much, and those who experienced greater levels of chewing or pronunciation discomfort. Again, similar patterns were seen in both sexes (Table 4).

### Correlation between depressive symptoms and subjective mastication ability and ability to pronounce

Participants were stratified by gender to analyze the following factors associated with depressive symptoms in the elderly: demographics, oral health, disease morbidity, health level, and health behavior. Males experiencing chewing difficulty had 1.45 times greater risk of experiencing depressive symptoms than males who did not experience chewing difficulty (95% CI, 1.29 to 1.63). Similarly, females experiencing chewing difficulty had 1.50 times greater risk of developing depressive symptoms than females reporting no chewing difficulties (95% CI, 1.39 to 1.61). Males who experienced discomfort with pronunciation had 1.97 times elevated risk of depressive symptoms than males who did not experience pronunciation discomfort (95% CI, 1.76 to 2.20). Females experiencing pronunciation discomfort had 1.55 times greater risk of depressive symptoms than females with no pronunciation discomfort (95% CI, 1.44 to 1.67).

In terms of age groups, across both sexes, the ‘younger’ group had 1.36 times greater risk of depressive symptoms than the ‘older’ group. Males and females with high school education or more were had 1.19 times (95% CI, 1.05 to 1.34) and 1.20 times (95% CI, 1.02 to 1.41) greater risk of developing depressive symptoms, respectively, than participants with elementary to middle school education. Males with no education had 1.70 times (95% CI, 1.48 to 1.95) greater risk of depressive symptoms, while female participants with no education had 1.55 times (95% CI, 1.32 to 1.82) greater risk of depressive symptoms than male and females with some education. Males receiving the NBLS benefits had a 2.57 times (95% CI, 2.23 to 2.96) greater risk of depressive symptoms than those who were not, while females had 1.95 times (95% CI, 1.79 to 2.12) greater risk of depressive symptoms.

In terms of level of health factors, the males and females with one to two chronic diseases had 1.27 times (95% CI, 1.14 to 1.42) greater risk and 1.19 times (95% CI, 1.09 to 1.29) greater risk of depressive symptoms, respectively, than their healthier counterparts. Males and females with three or more chronic diseases had 1.92 times greater risk, and 1.73 times greater risk of depressive symptoms, respectively, than their healthier counterparts. Stress level also influenced depressive symptoms risk. Males who experienced a greater levels of stress had 7.86 times (95% CI, 7.13 to 8.66) greater risk of depressive symptoms, whereas females had 6.53 times (95% CI, 6.13 to 6.97) greater risk of depressive symptoms than participants who experienced lower levels of stress or none at all.

Regarding health behavior, male current smokers had 1.11 times (95% CI, 0.97 to 1.26) greater risk of depressive symptoms than the males who had not smoked more than 100 cigarettes in their lifetime; whereas with female smokers, the risk was 1.16 times (95% CI, 1.02 to 1.33) greater. Among previous smokers, males had 0.94 (95% CI, 0.83 to 1.06) times greater risk of depressive symptoms, and females had 1.37 times (95% CI, 1.16 to 1.61) greater risk. In terms of drinking, males and females who have used alcohol within the previous year had a 1.31 times (95% CI, 1.14 to 1.50) and 1.18 times (95% CI, 1.08 to 1.29) greater risk of depressive symptoms than those who had never drunk alcohol. Sleep deprived (<6 hours) males and females participants had 1.66 times (95% CI, 1.49 to 1.85) and 1.57 times (95% CI, 1.47 to 1.68) greater risk of depressive symptoms, respectfully, than those who slept for six to eight hours. Among the participants whose daily sleeping hours exceeded eight hours (excluding hours spent napping during the day) males and females had 1.43 times (95% CI, 1.23 to 1.66) and 1.40 times (95% CI, 1.25 to 1.57) greater risk of depressive symptoms, respectfully (Table 5).

## DISCUSSION

In agreement with previous research, diminished ability to mastication or pronunciation was found to increases risk of depression in the elderly [14]. After adjusting for demographic characteristics, disease morbidity, health level, and health behavior, risk of depressive symptoms was 1.45 times greater (95% CI, 1.29 to 1.63) for males experiencing chewing difficulty compared with those who did not; findings consistent with that of a study using first-year data (2010) from the fifth National Health and Nutrition Examination Survey. They reported Korean adults experiencing greater levels of chewing difficulty having 1.25 times (95% CI, 1.05 to 1.50) greater risk of developing depression [15]. The current study found that females experiencing chewing difficulty had 1.50 times (95% CI, 1.39 to 1.61) greater risk of developing depressive symptoms than female with no chewing difficulties. The fact that females are at a greater risk suggests that there is a sex difference regarding the level of influence chewing difficulty exerts on depressive symptoms.

Males experiencing chewing difficulty had 1.97 (95% CI, 1.76 to 2.20) times greater risk of developing depressive symptoms than those who did not; while female participants reporting chewing difficulties had at a 1.55 times (95% CI, 1.44 to 1.67) greater risk of depressive symptoms than those who did not. These findings are supported by another study [16], which reported that clinical oral conditions exert a strong influence on depression. During one’s middle years, when social activity and opportunities for conversation are frequent, impaired speech may lead to depressive symptoms [17]. On the other hand, during later years, when one’s economic participation slows down and spare time increases, time spent eating increases. This change can trigger a sense of despair over their diminished oral function that was previously looked over, which can lead to depressive symptoms. The fact that males with chewing difficulties were found to be at greater risk of depressive symptoms than females appears to be attributable to the fact that males tend to participate in economic activity longer than most females. Improving the elderly’s mental health by helping them improve oral health appears to be an important task, which would ensure healthy senior years. As such, efforts must be made to provide diverse intervention programs designed to promote mental health and oral health in the elderly.

The current study has some limitations. First, because of its cross-sectional design, causal relationships cannot be inferred. Second, chronic diseases were defined as chronic conditions diagnosed by a doctor, perhaps introducing a selection bias. Third, because the CHS involved visiting sample households to conduct one-on-one interviews, individuals with severe conditions, such as long-term hospital and dementia patients, were excluded. Fourth, it is impossible to know whether participants depressive symptoms was being treated with medication. In addition, as the 2009 survey table, compiled while using the translated CES-D, was only included in the 2009 CHS data, the most recent data was not included. However, by comparing findings of other recent studies with the findings of the present study, efforts were made to overcome these limitations. Finally, mastication ability and pronunciation ability were evaluated subjectively, and nutrition-related variables (e.g., the number of fresh fruit/vegetables consumed), which can influence chewing and pronunciation ability, were not surveyed in 2009; this could have been examined further. However, despite such limitations, the present study is both representative and reliability as it used the CHS data; data collected nationwide from adults aged ≥19 years, from which a sample was systematically extracted.

Based on both the findings of the present study and previous studies, oral health and depressive symptoms in the elderly have a strong connection. If the increasing trend of depressive symptoms continues, programs designed to improve the elderly’s oral health and depressive symptoms management must be developed. Through comprehensive sustainable programs, individualized health education can be delivered to the elderly. Finally, efforts must be made to maintain and improve oral health through regular visits to dentists.

## Figures and Tables

**Table 1.** General characteristics of study participants by sex

		Male (n=20,582)		Female (n=30,112)	
		n	%^1^	n	%^1^
Age (yr)	65-74	14,420	70.1	18,997	63.1
	≥75	6,162	29.9	11,115	36.9
Educational level	High school and over	5,482	26.6	1,555	5.2
	Elementary-Middle school	10,913	53.0	10,852	36.0
	None	4,187	20.3	17,705	58.8
National Basic Livelihood Security	No	19,238	93.5	26,676	88.6
	Yes	1,344	6.5	3,436	11.4
No. of chronic diseases	0	7,345	35.7	5,539	18.4
	1-2	11,007	53.5	16,885	56.1
	≥3	2,230	10.8	7,688	25.5
Perceived stress	No	17,210	83.6	22,885	76.0
	Yes	3,372	16.4	7,227	24.0
Cigarette smoking	Never	5,125	24.9	27,759	92.2
	Past	9,399	45.7	893	3.0
	Current	6,058	29.4	1,460	4.9
Alcohol drinking	Never	4,102	19.9	19,391	64.4
	Past	5,132	24.9	4,006	13.3
	Current	11,348	55.1	6,715	23.3
Sleeping hours (hr)	6-8	14,904	72.4	20,219	67.2
	<6	3,794	18.4	7,650	25.4
	≥8	1,884	9.2	2,243	7.5
Chewing difficulty	No	10,510	51.1	13,464	44.7
	Yes	10,072	48.9	16,648	55.3
Pronunciation discomfort	No	15,304	74.4	21,633	71.8
	Yes	5,278	25.6	8,479	28.2
Depressive symptoms (CES-D)	No (<16)	17,966	87.3	23,097	76.7
	Yes (≥ 16)	20,582	12.7	7,015	23.3

1 Weighted percent was calculated for each respondent to approximate the national population with respect to age and sex, as defined in the 2005 census performed by the Korean National Statistical Office.

**Table 2.** Distribution of factors related to chewing difficulty by sex

	n^1^	Male (n=20,582)				n^1^	Female (n=30,112)			
		Weighted prevalence^2^		Age-adjusted prevalence^3^			Weighted prevalence^2^		Age-adjusted prevalence^3^	
		%	SE	%	95% CI		%	SE	%	95% CI
Educational level										
High school and over	5,482	35.8	0.6	34.6	32.8, 36.4	1,555	32.0	1.2	34.0	30.9, 37.1
Elementary-Middle school	10,913	50.7	0.5	48.9	47.5, 50.3	10,852	48.0	0.5	48.3	46.9, 49.7
None	4,187	61.6	0.8	57.8	55.4, 60.2	17,705	61.8	0.4	58.5	57.3, 59.7
National Basic Livelihood Security										
No	19,238	47.8	0.4	44.2	43.2, 45.2	26,676	53.4	0.3	50.5	49.7, 51.3
Yes	1,344	65.8	1.3	61.5	57.6, 65.4	3,436	69.8	0.8	65.9	63.5, 68.3
No. of chronic diseases										
0	7,345	47.1	0.6	43.5	41.9, 45.1	5,539	49.8	0.7	46.8	44.8, 48.8
1-2	11,007	48.9	0.5	44.6	43.2, 46.0	16,885	54.5	0.4	50.5	49.3, 51.7
≥3	2,230	55.2	1.1	52.3	49.4, 55.2	7,688	61.1	0.6	58.0	56.4, 59.6
Perceived stress										
No	17,210	46.1	0.4	42.0	41.0, 43.0	22,885	51.5	0.3	47.4	46.4, 48.4
Yes	3,372	63.4	0.8	60.4	58.0, 62.8	7,227	67.2	0.6	65.2	63.6, 66.8
Cigarette smoking										
Never	5,125	40.4	0.7	36.5	34.7, 38.3	27,759	54.2	0.3	51.2	50.4, 52.0
Past	9,399	49.5	0.5	44.3	42.9, 45.7	893	69.8	1.5	65.6	60.5, 70.7
Current	6,058	55.3	0.6	54.2	52.4, 56.0	1,460	67.4	1.2	61.8	57.9, 65.7
Alcohol drinking										
Never	4,102	49.2	0.8	43.8	41.6, 46.0	19,391	54.9	0.4	51.1	50.1, 52.1
Past	5,132	54.9	0.7	50.8	48.8, 52.8	4,006	61.3	0.8	58.2	56.0, 60.4
Current	11,348	46.2	0.5	43.5	42.1, 44.9	6,715	52.9	0.6	50.5	48.7, 52.3
Sleeping hours (hr)										
6-8	14,904	46.6	0.4	43.1	41.9, 44.3	10,638	52.6	0.4	49.6	48.6, 50.6
<6	3,794	54.9	0.8	50.7	48.3, 53.1	4,684	61.2	0.6	57.3	55.7, 58.9
≥8	1,844	55.1	1.1	51.4	47.9, 54.9	1,326	59.1	1.0	55.3	52.0, 58.6

SE, standard error; CI, confidence interval.

1 Number of participants.

2 Weighted prevalence was calculated for each respondent to approximate the national population with respect to age and sex, as defined in the 2005 census performed by the Korean National Statistical Office.

3 Age-adjusted prevalence was calculated by the direct method and the age-distribution for the 2005 census population.

**Table 3.** Distribution of factors related to pronunciation discomfort by sex

	n^1^	Male (n=20,582)				n^1^	Female (n=30,112)			
		Weighted prevalence^2^		Age-adjusted prevalence^3^			Weighted prevalence^2^		Age-adjusted prevalence^3^	
		%	SE	%	95% CI		%	SE	%	95% CI
Educational level										
High school and over	5,482	17.0	0.5	15.8	14.6, 17.0	1,555	13.7	0.9	14.4	12.0, 16.8
Elementary-Middle school	10,913	25.9	0.4	24.4	23.2, 25.6	10,852	21.9	0.4	22.0	20.8, 23.2
None	4,187	36.3	0.7	32.2	29.8, 34.6	17,705	33.2	0.4	30.3	29.3, 31.3
National Basic Livelihood Security										
No	19,238	24.5	0.3	21.7	20.9, 22.5	26,676	26.6	0.3	24.2	23.4, 25.0
Yes	1,344	41.5	1.3	37.8	34.1, 41.5	3,436	40.6	0.8	37.8	35.4, 40.2
No. of chronic diseases										
0	7,345	24.0	0.5	20.8	19.4, 22.2	5,539	27.1	0.6	24.0	22.4, 25.6
1-2	11,007	25.8	0.4	22.5	21.5, 23.5	16,885	27.4	0.3	24.3	23.3, 25.3
≥3	2,230	30.5	1.0	27.6	25.1, 30.1	7,688	30.5	0.5	28.8	27.4, 30.2
Perceived stress										
No	17,210	23.4	0.3	20.1	19.3, 20.9	22,885	26.0	0.3	22.9	22.1, 23.7
Yes	3,372	36.9	0.8	34.7	32.5, 36.9	7,227	35.1	0.6	33.4	31.8, 35.0
Cigarette smoking										
Never	5,125	23.1	0.6	19.2	17.8, 20.6	27,759	27.3	0.3	24.8	24.0, 25.6
Past	9,399	25.3	0.4	21.7	20.5, 22.9	893	37.0	1.6	33.6	28.9, 38.3
Current	6,058	28.3	0.6	27.0	25.4, 28.6	1,460	39.2	1.3	36.5	33.0, 40.0
Alcohol drinking										
Never	4,102	28.8	0.7	24.6	22.8, 26.4	19,391	29.1	0.3	26.0	25.2, 26.8
Past	5,132	32.3	0.7	28.6	26.8, 30.4	4,006	31.1	0.7	29.3	27.3, 31.3
Current	11,348	21.5	0.4	19.5	18.5, 20.5	6,715	23.6	0.5	21.5	20.1, 22.9
Sleeping hours (hr)										
6-8	14,904	23.8	0.3	20.8	19.8, 21.8	20,219	26.7	0.3	24.2	23.4, 25.0
<6	3,794	29.5	0.7	26.5	24.5, 28.5	7,650	30.3	0.5	27.8	26.4, 29.2
≥8	1,884	32.3	1.1	28.5	25.6, 31.4	2,243	34.2	1.0	28.4	25.9, 30.9

SE, standard error; CI, confidence interval.

1 Number of participants.

2 Weighted prevalence was calculated for each respondent to approximate the national population with respect to age and sex, as defined in the 2005 census performed by the Korean National Statistical Office.

3 Age-adjusted prevalence was calculated by the direct method and the age-distribution for the 2005 census population.

**Table 4.** Distribution of factors related to depressive symptoms by sex

	n^1^	Male (n=20,582)				n^1^	Female (n=30,112)			
		Weighted prevalence^2^		Age-adjusted prevalence^3^			Weighted prevalence^2^		Age-adjusted prevalence^3^	
		%	SE	%	95% CI		%	SE	%	95% CI
Educational level										
High school and over	5,482	9.3	0.4	10.1	8.9, 11.3	1,555	14.6	0.9	16.2	12.0, 16.8
Elementary-Middle school	10,913	12.0	0.3	13.3	12.3, 14.3	10,852	19.2	0.4	21.5	20.8, 23.2
None	4,187	18.9	0.6	20.6	18.4, 22.8	17,705	26.6	0.3	29.6	29.3, 31.3
National Basic Livelihood Security										
No	19,238	11.3	0.2	11.8	11.2, 12.4	26,676	20.8	0.2	22.4	23.4, 25.0
Yes	1,344	32.6	1.3	35.5	31.8, 39.2	3,436	42.6	0.8	48.1	35.4, 40.2
No. of chronic diseases										
0	7,345	9.7	0.3	10.2	9.2, 11.2	5,539	16.9	0.5	17.8	22.4, 25.6
1-2	11,007	13.0	0.3	13.5	12.5, 14.5	16,885	21.4	0.3	22.6	23.3, 25.3
≥3	2,230	21.2	0.9	19.4	17.2, 21.6	7,688	32.1	0.5	33.7	27.4, 30.2
Perceived stress										
No	17,210	7.2	0.2	7.4	6.8, 8.0	22,885	13.7	0.2	14.6	22.1, 23.7
Yes	3,372	41.0	0.8	41.5	39.1, 43.9	7,227	53.8	0.6	55.4	31.8, 35.0
Cigarette smoking										
Never	5,125	11.0	0.4	10.7	9.5, 11.9	27,759	22.5	0.3	24.1	24.0, 25.6
Past	9,399	12.7	0.3	13.0	12.0, 14.0	893	35.3	1.6	37.5	28.9, 38.3
Current	6,058	14.3	0.4	15.7	14.3, 17.1	1,460	31.8	1.2	36.4	33.0, 40.0
Alcohol drinking										
Never	4,102	12.8	0.5	13.2	11.6, 14.8	19,391	23.2	0.3	24.4	25.2, 26.8
Past	5,132	18.9	0.5	19.4	17.8, 21.0	4,006	29.6	0.7	31.5	27.3, 31.3
Current	11,348	9.9	0.3	10.5	9.7, 11.3	6,715	20.0	0.5	22.4	20.1, 22.9
Sleeping hours (hr)										
6-8	14,904	10.3	0.2	10.8	10.0, 11.6	20,219	19.0	0.3	20.8	23.4, 25.0
<6	3,794	20.3	0.7	20.7	18.9, 22.5	7,650	33.8	0.5	34.6	26.4, 29.2
≥8	1,884	16.2	0.8	16.9	14.2, 19.6	2,243	26.2	0.9	26.7	25.9, 30.9
Chewing difficulty										
No	10,510	7.1	0.3	7.6	6.8, 8.4	13,464	14.6	0.3	16.1	15.1, 17.1
Yes	10,072	18.5	0.4	19.9	18.7, 21.1	16,648	30.3	0.4	33.3	32.3, 34.3
Pronunciation discomfort										
No	15,304	8.8	0.2	9.2	8.6, 9.8	21,633	18.8	0.3	20.5	19.7, 21.3
Yes	5,278	24.2	0.6	26.8	24.8, 28.8	8,479	34.9	0.5	38.3	36.7, 39.9

SE, standard error; CI, confidence interval.

1 Number of participants.

2 Weighted prevalence was calculated for each respondent to approximate the national population with respect to age and sex, as defined in the 2005 census performed by the Korean National Statistical Office.

3 Age-adjusted prevalence was calculated by the direct method and the age-distribution for the 2005 census population.

**Table 5.** The relationship between subjective oral mastication ability, pronunciation ability condition and depressive symptoms in the elderly

		Male (n=20,582)				Female (n=30,112)			
		Model 1	Model 2	Model 3	Model 4	Model 1	Model 2	Model 3	Model 4
Chewing difficulty	No	1.00 (reference)	1.00 (reference)	1.00 (reference)	1.00 (reference)	1.00 (reference)	1.00 (reference)	1.00 (reference)	1.00 (reference)
	Yes	1.93 (1.73, 2.15)	1.78 (1.60, 1.99)	1.48 (1.32, 1.66)	1.45 (1.29, 1.63)	2.02 (1.89, 2.16)	1.87 (1.75, 2.00)	1.54 (1.43, 1.65)	1.50 (1.39, 1.61)
	p-value	<0.001	<0.001	<0.001	<0.001	<0.001	<0.001	<0.001	<0.001
Pronunciation discomfort	No	1.00 (reference)	1.00 (reference)	1.00 (reference)	1.00 (reference)	1.00 (reference)	1.00 (reference)	1.00 (reference)	1.00 (reference)
	Yes	2.30 (2.08, 2.54)	2.12 (1.91, 2.35)	2.04 (1.83, 2.28)	1.97 (1.76, 2.20)	1.62 (1.51, 1.72)	1.52 (1.42, 1.62)	1.55 (1.45, 1.67)	1.55 (1.44, 1.67)
	p-value	<0.001	<0.001	<0.001	<0.001	<0.001	<0.001	<0.001	<0.001
Age (yr)	65-74		1.00 (reference)	1.00 (reference)	1.00 (reference)		1.00 (reference)	1.00 (reference)	1.00 (reference)
	≥75		1.22 (1.11, 1.33)	1.44 (1.30, 1.59)	1.36 (1.28, 1.50)		1.08 (1.02, 1.15)	1.42 (1.33, 1.51)	1.36 (1.28, 1.45)
	p-value		<0.001	<0.001	<0.001		0.008	<0.001	<0.001
Educational level	High school and over		1.00 (reference)	1.00 (reference)	1.00 (reference)		1.00 (reference)	1.00 (reference)	1.00 (reference)
	Elementary-Middle school		1.09 (0.98, 1.22)	1.20 (1.07, 1.36)	1.19 (1.05, 1.34)		1.19 (1.02, 1.39)	1.19 (1.01, 1.40)	1.20 (1.02, 1.41)
	p-value		0.009	0.04	0.05		0.55	0.35	0.46
	None		1.50 (1.32, 1.70)	1.75 (1.52, 2.01)	1.70 (1.48, 1.95)		1.49 (1.29, 1.73)	1.55 (1.32, 1.82)	1.55 (1.32, 1.82)
	p-value		<0.001	<0.001	<0.001		<0.001	<0.001	<0.001
National Basic Livelihood Security	No		1.00 (reference)	1.00 (reference)	1.00 (reference)		1.00 (reference)	1.00 (reference)	1.00 (reference)
	Yes		3.01 (2.65, 3.42)	2.72 (2.36, 3.13)	2.57 (2.23, 2.96)		2.41 (2.23, 2.60)	1.02 (1.86, 2.20)	1.95 (1.79, 2.12)
	p-value		<0.001	<0.001	<0.001		<0.001	<0.001	<0.001
No. of chronic diseases	0			1.00 (reference)	1.00 (reference)			1.00 (reference)	1.00 (reference)
	1-2			1.29 (1.16, 1.43)	1.27 (1.14, 1.42)			1.19 (1.09, 1.30)	1.19 (1.09, 1.29)
	p-value			0.05	0.08			<0.001	0.001
	≥3			2.00 (1.73, 2.31)	1.92 (1.66, 2.23)			1.78 (1.62, 1.96)	1.73 (1.58, 1.91)
	p-value			<0.001	<0.001			<0.001	<0.001
Perceived stress	No			1.00 (reference)	1.00 (reference)			1.00 (reference)	1.00 (reference)
	Yes			1.29 (1.16, 1.43)	7.86 (7.13, 8.66)			6.89 (6.47, 7.34)	6.53 (6.13, 6.97)
	p-value			<0.001	<0.001			<0.001	<0.001
Cigarette smoking	Never				1.00 (reference)				1.00 (reference)
	Past				0.94 (0.83, 1.06)				1.37 (1.16, 1.61)
	p-value				0.02				0.006
	Current				1.11 (0.97, 1.26)				1.16 (1.02, 1.33)
	p-value				0.01				0.92
Alcohol drinking	Never				1.00 (reference)				1.00 (reference)
	Past				1.31 (1.14, 1.50)				1.18 (1.08, 1.29)
	p-value				<0.001				<0.001
	Current				0.78 (0.69, 0.89)				0.86 (0.79, 0.93)
	p-value				<0.001				<0.001
Sleeping hours (hr)	6-8				1.00 (reference)				1.00 (reference)
	<6				1.66 (1.49, 1.85)				1.57 (1.47, 1.68)
	p-value				<0.001				<0.001
	≥8				1.43 (1.23, 1.66)				1.40 (1.25, 1.57)
	p-value				0.18				0.04

Values are presented as odds ratio (95% confidence interval).

## References

[1] Suh UJ, Lee JY (2010). Estimated future population (2010-2060).

[2] Han DH (2013). Chewing difficulty and multiple chronic conditions in Korean elders: KNHANES IV. J Korean Dent Assoc.

[3] Health Insurance Review & Assessment Service (2014). Reviewed medical fee.

[4] Lee HL, Lee HM, Kim HJ, Oh KW (2015). Trends in oral health status among adults over 65 years old in Korea, 2007-2013. Public Health Wkly Rep.

[5] Kim SK, Kwon MY (2006). Analysis of satisfaction level regarding removable dentures for aged patients in dental hospitals or clinics. J Korean Acad Dent Hyg Educ.

[6] Oh JK, Kim YJ, Kho HS (2001). A study on the clinical characteristics of patients with dry mouth. Korean J Oral Med.

[7] Kim YA (2014). Depression symptoms experience among adults in Korea, 2012. Public Health Wkly Rep.

[8] Lee HS, Kim C (2012). Effects of oral health impact profile (OHIP) on depression and quality of life among community-dwelling Korean elderly persons. J Korean Acad Community Health Nurs.

[9] Kim KW (2014). The effects of oral exercise on xerostomia and depression scale among the elderly patients. Korean Acad Dent Hyg.

[10] Bojorquez-Chapela I, Villalobos-Daniel VE, Manrique-Espinoza BS, Tellez-Rojo MM, Salinas-Rodríguez A (2009). Depressive symptoms among poor older adults in Mexico: prevalence and associated factors. Rev Panam Salud Publica.

[11] Takiguchi T, Yoshihara A, Takano N, Miyazaki H (2015). Oral health and depression in older Japanese people.

[12] Radloff LS (1977). The CES-D scale a self-report depression scale for research in the general population. Appl Psychol Meas.

[13] Cho MJ, Kim KH (1993). Diagnostic validity of the CES-D (Korean version) in the assessment of DSM-III-R major depression. J Korean NeuropsychiatrAssoc.

[14] Lee MA (2011). Relative effects of health and family factors on geriatric depression. Korean J Community Living Sci.

[15] Park SJ, Ko KD, Shin SI, Ha YJ, Kim GY, Kim HA (2014). Association of oral health behaviors and status with depression: results from the Korean National Health and Nutrition Examination Survey, 2010. J Public Health Dent.

[16] Jang JH, Kin SH (2007). The relationship between xerostomia and depression in elderly people. Korean J Health Educ Promot.

[17] Lee HJ, Kahng SK, Lee JY (2008). The effects of socioeconomic position and health behavior on geriatric depressive symptom. J Korean Gerontol Soc.

