# Supplementary material for: Correlation between depressive symptoms and subjective mastication ability and ability to pronunciation among Korean elderly
Source: Epidemiol Health. 2016 Jul 25;38:e2016035. doi: 10.4178/epih.e2016035 (PMC5063820; doi:10.4178/epih.e2016035)
Supplement: Supplementary file 1 [file epih-38-e2016035-supplementary.pdf]

The Relationship Between Oral Health Behavior and Depressive Symptoms in the Korean Elderly

박민선[1)2)], 황경균[3)], 최보율[1)2)4)]

한양대학교 건강과 사회연구소[1)], 한양대학교 일반대학원 보건학과[2)], 한양대학교 의과대학 치과학교실[3)], 한양대학교 의과대학 예방의학교실[4)]

교신저자: 최보율

주소: (04763) 서울특별시 성동구 왕십리로 222 한양대학교 의과대학 예방의학교실

전화: +82-2-2220-0662 팩스: +82-2-2293-0660

E-mail: bychoi@hanyang.ac.kr

한국 노인의 구강건강상태와 우울증상과의 관련성

목적: 이 연구의 목적은 노인의 구강건강과 우울증상과의 관련성을 살펴봄으로써 노인을 대상으로 구강건강 관리 중재 프로그램의 개발을 위한 기초자료를 제공하고자 하는데 있다.

방법: 지역사회의 일반 인구를 대상으로 우울증 정도를 측정하는 데 있어 세계적으로 가장 널리 사용되는 조사도구인 The Center for Epidemiologic Studies-Depression Scale(CES-D)을 번역하여 조사된 2009년 지역사회건강조사 데이터를 이용하여 결측치를 제외한 만 65세 이상 노인 50,694명(남성 20,582명, 여성 30,112명)을 최종 연구대상으로 하였다. CES-D 16점 이상을 우울증상이 있는 것으로 정의하였으며, 데이터 분석은 SAS version 9.3을 사용하여 실행하였다.

결과: 사회경제적 수준이 낮을수록, 복합만성질환자일수록, 건강수준이 낮을수록, 건강행태가 좋지 않을수록, 저작 및 발음불편을 호소할수록 우울증상 유병률이 높게 나타났다. 남성 노인에서 저작불편을 호소하지 않는 그룹에 비해 저작불편을 호소하는 그룹에서 우울증상을 겪을 가능성이 1.45배(95% CI:1.29-1.63) 높았으며, 발음불편을 호소하지 않는 그룹에 비해 발음불편을 호소하는 그룹에서 우울증상을 겪을 가능성이 1.97배(95% CI:1.76-2.20) 높았다. 여성 노인에서 저작불편을 호소하지 않는 그룹에 비해 저작불편을 호소하는 그룹에서 우울증상을 겪을 가능성이 1.50배(95% CI:1.39-1.61) 높았으며, 발음불편을 호소하지 않는 그룹에 비해 발음불편을 호소하는 그룹에서 우울증상을 겪을 가능성이 1.55배(95% CI:1.44-1.67) 높았다.

결론: 노인의 성별에 따른 구강건강 관리와 우울증상 관리를 중재하는 프로그램의 개발 및 접근이 필요하며, 지역별로 유병률이 상이할 수 있기 때문에 시ㆍ군ㆍ구 소지역 단위를 고려한 추가 연구가 필요할 것으로 보인다.

주제어 : 노인, 구강건강, 우울증상, 관련성

한국 노인의 구강건강상태와 우울증상과의 관련성

서론

2011년 통계청 자료에 의하면 출산율이 낮아지고 평균수명이 늘어나면서 인구피라미드는 점차 아랫부분이 좁아지고, 윗부분(특히 고령)이 넓어지는 역삼각형의 항아리 구조로 변화될 것으로 전망하고 있다[1]. 노인은 기능상 장애 및 상실과 신체활동의 변화 등에 의한 와상상태, 장기간의 섭생 및 영양문제, 완치불가능한 만성질병으로 인해 다른 연령층과는 다른 특징을 보인다[2]. 2014년 건강보험심사평가원 자료에 의하면 65세 이상 노인인구 구성비는 11.9%로 전년대비 0.4% 증가하였으며, 진료비 구성비도 35.5%로 전년대비 1.0% 증가하였다. 노인 환자의 외래 진료비 증가율이 가장 높은 상병은 치은염 및 치주질환으로 연간 1,223억원(30.1% 증가)이었으며, 환자 1명당 진료비는 6만 8천원으로 나타났다[3].

구강건강은 사람의 건강상태를 평가하는데 있어 가장 기본적인 척도로, 특히 치아가 불량하면 음식물 섭취가 곤란하여 건강을 위협할 수 있다. 구강 및 치아는 저작, 심미, 발음과 같은 일상생활에서 중요한 기능을 수행하므로, 구강건강을 제외하고는 건강을 논할 수가 없다. 2015년 질병관리본부 자료에 의하면 65세 이상 노인 중 20개 이상 자연치아를 보유하지 못한 사람이 절반에 달하였으며, 임플란트 치료의 비율이 증가하는 등 상실된 치아의 기능을 복구하기 위한 치료가 동반되고 있지만 아직 노인의 절반 정도가 씹는 데 불편함을 경험하고 있었다[4]. 치아를 상실하게 되면 저작능력이 저하되어 섭취할 수 있는 음식물의 선택 범위가 좁아지게 되고 이로 인해 식사의 양과 질이 저하됨으로써 건강을 유지하기가 어려워질 수 있다[5]. 노인층이 증가하면서 구강건조증 및 이와 관련된 증상을 호소하는 환자가 증가하고 있는데, 타액의 분

비가 감소되면 구강과 구순 및 인후의 건조감으로 인해 발음을 할 때, 자극적인 음식의 저작과 연하시, 구강점막의 통증 등과 같은 기능적 불편감 뿐만 아니라, 타액의 자정작용의 감소로 인해 구취 및 치아우식증이나 치주질환이 증가하고, 구강 캔디다증과 같은 진균성 기회감염이 증가할 가능성이 있다[6]. 따라서 노인의 구강건강 수준을 개선하기 위한 지속적인 노력이 필요할 것으로 보인다.

2012년 국민건강영양조사 결과에 의하면 우울감 경험률은 최근 1년 동안 연속적으로 2주 이상 일상생활에 지장이 있을 정도의 슬픔이나 절망감 등을 경험한 것으로 남성보다 여성에서 우울감 경험률이 1.8배 높게 나타났으며, 연령과 함께 증가하여 70세 이상에서 가장 높게 나타났다[7].

노인에서 구강건강과 우울증상의 상관관계를 분석하는 것은 향후 노인인구에서 구강건강과 관련된 우울증상을 관리하는 데 있어 필요한 일이라고 할 수 있다. 국내 일부 지역 경로당 노인의 구강건강 영향지수(Oral Health Impact Profile-14, OHIP-14)는 우울(Geriatric Depression Scale-Short Form, GDS-SF)과 삶의 질(Quality of Life, QOL)과 밀접한 상관성을 가지고 있었으며[8], 일부 지역 일개 보건지소 구강체조프로그램에 참가한 노인의 우울척도(Short-form Geriatric Depres-sion Scale, SGDS-K) 점수는 구강체조프로그램 참여 전에 비해 참여 후에 낮아졌다고 보고되고 있으나[9], 일부지역에 한정된 연구여서 일반화시키기에는 어려움이 따른다. 한국 성인에서 우울증이 구강건강상태, 구강건강 행태(주관적 건강상태, 현재 저작불편, 치통 경험, 현재 턱관절장애 증상)와 의미있는 연관성이 있다는 보고가 있으나, 노인을 만 19세 이상 성인에 포함시켜 생애주기별 특징을 충분히 반영하지 못하였고, 일생동안 우울증 경험 여부를 묻는 단일문항에 대한 응답을 평생 우울증이라고 정의하는 등 노인인구의 특성을 연구에서 충분히 반영되지는 못하였다. 국외 연구로는 독일 출생자 코호트 자료를 이용하여 구강건강(Geriatric Oral Health Assessment Index, GOHAI)과 우울증(Self-rating Depression Scale, SDS)과의 관련성이 있었다고 보고되고 있으며[10], 일본일부 지역사회 거주 노인의 비자극 타액 유량, 구강 통증, 저작불편이 우울증과 관련이 있었다고 보고되고 있다[11]. 그러나 나라별로 문화에 따른 차이가 있을 수 있고, 생애주기별로 차이가 있을 수 있기 때문에

한국 노인에서도 비슷한 결과를 보일 것이라고 일반화시키기에는 자료가 충분하지 않았다. 이와 같이 국내에서도 노인의 구강건강이 우울에 미치는 영향에 관한 연구들이 최근 보고되고 있으나[12][13] 일부지역에 한정된 연구였으며, 연구에 사용된 우울척도 또한 다양하여 일반화시키기에는 어려움이 있었다. 본 연구에서는 노인인구가 급속도로 증가하고 있는 가운데 노인의 구강건강과 우울증상과의 관련성을 살펴봄으로써 노인의 구강건강 관리와 우울증상 관리를 중재하는 프로그램의 개발 및 접근에 있어 기초자료를 제공하고자 한다. 또한 성별에 따라 우울증상과 저작 및 발음불편 호소감이 다를 수 있으므로 성별로 층화하여 구강건강과 우울증상을 살펴보고자 한다.

연구대상 및 방법

1. 연구대상 및 조사방법

본 연구는 질병관리본부 주체로 2009년 9월부터 11월까지 3개월 간 전국의 253개 보건소와 35개 대학교가 협약을 체결하여 수행된 '2009년 지역사회 건강조사' 종이조사표 면접조사(PAPI : Paper Assisted Personal Interviewing) 원시자료를 이용하였다.

지역사회건강조사의 조사대상은 만 19세 이상 성인으로 2008년부터 시행되었으며, 표본으로 선정된 가구에 훈련된 조사원이 방문하여 1대1 면접조사로 진행하며, 매년 보건소별로 평균 900명을 조사한다. 재개발 혹은 재건축으로 거주가 불가능한 지역, 상업 혹은 공단 밀집지역으로 거주가 희박한 지역, 특정집단(한센촌, 기숙사, 종교단체집단거주지) 거주지역 등은 표본가구에서 제외하였으며, 1대1 면접조사임을 감안하여 3회 이상 접촉이 불가능하거나 타지역 및 해외 장기체류, 장기입원 등의 이유로 조사기간 내에 접촉 불가능한 가구원은 조사대상에서 제외하였다(지역사회건강조사 원시자료 이용지침서, 2009).

본 연구에서는 지역사회의 일반 인구를 대상으로 우울증 정도를 측정하는 데 있어 세계적으로 가장 널리 사용되는 조사도구인 The Center for Epidemiologic Studies-Depression Scale(CES-D)을 한국어로 번역하여 사용한 조사표가 있는 2009년 지역사회건강조사 데이터를 이용하여 조사가 완료된 전체 대상자

총 230,725명 중 만 65세 이상 노인 50,694명(남성 20,582명, 여성 30,112명)을 최종 연구대상으로 하였다.

2. 조사도구

1) 노인

2009년 기준 1934년 7월 31일 이후 ~ 1944년 7월 31일 이전 출생자의 경우 '만 65세 이상~만 74세 이하' 를 전기노인, 1934년 7월 31일 이전 출생자의 경우 '만 75세 이상' 을 후기노인으로 분류하였다.

2) 구강 건강

구강건강은 상병에 이환되어 있지 않고 정신작용과 사회생활에 장애가 되지 않는 악안면구강 조직기관의 상태로 저작불편과 발음불편으로 평가하였다. 2009년 지역사회건강조사에서 저작불편 호소여부에 대해 현재 치아나 틀니, 잇몸 등 입안의 문제로 음식을 씹는 데 어려움이나 불편감을 느끼십니까?(※틀니를 사용하시는 경우에는 틀니를 낀 상태에서 느끼는 상태를 말씀해 주십시오)라는 문항으로 질문을 하였으며, 발음불편 호소여부는 현재 치아나 틀니, 잇몸 등 입안의 문제로 명확히 발음을 하는 데에 어려움이나 불편감을 느끼십니까?(※틀니를 사용하시는 경우에는 틀니를 낀 상태에서 느끼는 상태를 말씀해 주십시오)라는 문항으로 질문을 하였다. 이에 대한 응답으로 '전혀 불편하지 않다' 와 '별로 불편하지 않다' , '그저 그렇다' 는 '아니오' 로 '불편하다' 와 '매우 불편하다' 는 '예' 로 분류하였다.

3) 우울증상

우울은 Radloff(1977)에 의해 개발되고, Cho와 Kim(1993)에 의해 번역[14]되어 신뢰도와 타당도가 검증된 The Center for Epidemiologic Studies Depression (CES-D) Scale 한국어판을 이용하여 측정하였다. CES-D는 자기 보고형 우울증 간이 선별검사 도구의 하나로 20개 문항으로 이루어져 있으며, 각 문항에 대해 지난 일주일간 전혀, 거의 없음(1일 이하)은 0점, 약간 있음(1~2일) 1점, 가끔/종종(3~4일) 2점, 대부분/항상(5일 이상) 3점으로 총 점수의 범위는 0~60점이며, 점수가 높을수록 우울 수준이 높고, Cho와 Kim (1993)이 제시한 21점을 기준으로 21점 이상이면 우울이 있는 것으로 보았다.

4) 인구사회학적 요인, 질병이환, 건강수준 및 건강행태 요인

성별을 층화하여 ‘남성’과 ‘여성’으로 분류하였으며, 연령은 2009년 기준 1934년 7월 31일 이후 ~ 1944년 7월 31일 이전 출생자의 경우 ‘만 65세 이상~만 74세 이하’, 1934년 7월 31일 이전 출생자의 경우 ‘만 75세 이상’으로 분류하였다. 교육수준은 ‘무학’, ‘초등학교~중학교 졸업’, ‘고등학교 졸업 이상’으로 분류하였으며, 현재 기초생활 수급자인 경우 ‘예’, 기초생활 수급자가 아닌 경우와 과거 수급자의 경우 ‘아니오’로 분류하였다.
만성질환 이환 수는 고혈압, 당뇨병, 고지혈증, 뇌졸중, 심근경색, 협심증, 관절염, 골다공증, 폐결핵, 천식, 비염 중 의사진단을 받은 질병의 개수로 0개, 1~2개, 3개 이상으로 분류하였으며, 주관적 스트레스 수준은 평소 일상생활 중 느끼는 스트레스 수준을 묻는 문항에 ‘거의 느끼지 않는다’, ‘조금 느끼는 편이다’라고 응답한 경우 ‘아니오’로, ‘많이 느끼는 편이다’, ‘대단히 느낀다’라고 응답한 경우 ‘예’로 분류하였다.
현재 담배를 피우는지에 대한 응답은 ‘비해당’의 경우 ‘비흡연’으로 ‘과거에는 피웠으나 현재 피우지 않음’의 경우 ‘과거 흡연’, ‘매일 피움’과 ‘가끔 피움’의 경우 ‘현재 흡연으로 분류하였다. 최근 1년 동안 술을 마신 적이 있는지에 대한 응답은 ‘비해당’의 경우 ‘비음주’로 ‘예’라고 응답한 경우 ‘현재 음주’로 ‘아니오’라

고 응답한 경우 ‘ 과거 음주’로 분류하였다. 수면시간은 낮잠시간을 제외한 하루 평균 수면시간을 의미하며, 사사분위로 ‘6시간 미만’, ‘6~8시간 미만’, ‘8시간 이상’으로 구분하였다.

3. 자료분석

노인의 일반적 특성을 살펴보기 위하여 기술분석을 실시하였으며, 저작불편 호소율, 발음불편 호소율, 우울증상 유병률에 영향을 미치는 요인을 살펴보기 위해 chi-square test를 실시하였다. 노인의 구강건강과 우울증상과의 관련성을 파악하기 위해 다중로지스틱회귀분석을 수행하였으며, 인구사회학적, 질병이환, 건강수준, 건강행태 관련 변수를 보정하여 비차비(adjusted odds ratio : Adjusted OR)와 95% 신뢰구간(95% confidence interval: 95% CI)를 산출하였다. 성별로 층화하여 분석하였으며, 수면시간은 평균 수면시간을 사사분위(quarterlies)로 구분하여 분석하였다. 모든 통계처리는 SAS Ver 9.3을 이용하였다.

연구결과

1. 대상자의 일반적 특성

만 65세 이상 노인의 성별분포를 살펴보면 남성 노인에 비해 여성 노인의 비율이 높았다.

남성의 경우 저작불편을 호소하는 군은 48.9%, 발음불편을 호소하는 군은 25.6%로 발음불편을 호소하는 경우에 비해 저작불편을 호소하는 경우가 더 많았다. 우울증상을 겪고 있는 군(CES-D 16점 이상)은 12.7%였다. 후기 노인에 비해 전기노인의 비율이 더 높게 나타났으며, 교육수준은 중학교 졸업 이하인 경우가 73.3%를 차지하였다. 현재 기초생활수급자라고 응답한 비율은 6.5%였다. 만성질환을 1개 이상 보유하고 있는

경우가 절반 이상이었으며, 만성질환을 3개 이상 보유한 복합만성질환자의 분율은 10.8%였다. 주관적 스트레스는 비교적 스트레스를 적게 느끼는 군의 비율이 높았다. 비흡연자에 비해 흡연경험자의 비율이 높았으며, 흡연 경험자 중 과거 흡연자의 비율이 높게 나타났다. 음주 경험자 중 현재 음주자의 비율이 가장 높게 나타났으며, 하루 평균 수면시간은 수면 부족 또는 수면 과다증이 의심되는 경우가 27.6%를 차지하였다(Table 1).

여성의 경우 저작불편을 호소하는 군은 55.3%, 발음불편을 호소하는 군은 28.2%로 발음불편을 호소하는 경우에 비해 저작불편을 호소하는 경우가 더 많았다. 우울증상을 겪고 있는 군(CES-D 16점 이상)은 23.3%였다. 남성과 마찬가지로 후기 노인에 비해 전기노인의 비율이 더 높게 나타났으며, 교육수준은 중학교 졸업 이하인 경우가 94.8%를 차지하였다. 현재 기초생활수급자라고 응답한 비율은 11.4%였다. 만성질환을 1개 이상 보유하고 있는 경우가 절반 이상이었으며, 만성질환을 3개 이상 보유한 복합만성질환자의 분율은 25.5%였다. 주관적 스트레스는 비교적 스트레스를 적게 느끼는 군의 비율이 높았다. 흡연 경험자에 비해 비흡연자의 비율이 높았으며, 음주 경험자에 비해 비음주자의 비율이 가장 높게 나타났다. 하루 평균 수면시간은 수면 부족 또는 수면 과다증이 의심되는 경우가 32.9%를 차지하였다(Table 1).

2. 저작불편 호소율에 영향을 미치는 주요요인

인구사회학적 특성에 따른 연령-보정 저작불편 호소율은 교육수준이 낮을수록 높게 나타났으며, 기초생활수급자인 경우가 아닌 경우에 비해 저작불편 호소율이 더 높았다. 건강수준에 따른 저작불편 호소율은 만성질환 보유개수가 많을수록 높게 나타났으며, 스트레스가 많을수록 높게 나타났다. 건강행태에 따라서는 남성의 경우 흡연 경험자 중 현재 흡연자에서 저작불편 호소율이 높게 나타났다. 음주 경험자 중 과거 음주자에서 저작불편 호소율이 높게 나타났으며, 수면 부족 또는 수면 과다인 경우에도 저작불편 호소율이 높게 나타났으며, 남녀 모두 비슷한 양상을 보였다(Table 2).

3. 발음불편 호소율에 영향을 미치는 주요요인

인구사회학적 특성에 따른 연령-보정 발음불편 호소율은 남녀 모두 교육수준이 낮을수록 높게 나타났으며, 기초생활수급자일 경우에 발음불편 호소율이 높게 나타났다. 건강수준에 따른 발음불편 호소율은 만성질환 보유개수가 많을수록, 스트레스가 많을수록 높게 나타났다. 건강행태에 따라서는 남녀 모두 흡연 경험자 중 현재 흡연자에서 발음불편 호소율이 높게 나타났으며, 음주경험자 중 과거 음주자에서 저작불편 호소율이 높게 나타났다. 수면 부족 또는 수면 과다인 경우에도 발음불편 호소율이 높게 나타났으며, 남녀 모두 비슷한 양상을 보였다(Table 3).

4. 우울증상 유병률에 영향을 미치는 주요요인

연령-보정 우울증상 유병률은 남녀 모두 교육수준이 낮을수록 높게 나타났으며, 기초생활수급자일 경우에 높게 나타났다. 또한 건강수준에 따른 우울증상 유병률은 만성질환 보유개수가 많을수록, 스트레스가 많을수록 우울증상 유병률이 높게 나타났으며, 건강행태에 따라서는 남녀 모두 흡연경험자 중 현재 흡연자에서 음주 경험자 중 과거 음주자에서 우울증상 유병률이 높게 나타났다. 수면 부족 또는 수면 과다인 경우에도 우울증상 유병률이 높게 나타났으며, 저작이 불편한 경우와 발음이 불편한 경우 모두 우울증상 유병률이 높게 나타났다. 이는 남녀 모두에서 비슷한 양상을 보였다(Table 4).

5. 구강건강과 우울증상과의 관련성

노인의 우울증상과 관련된 인구사회학적 요인 및 구강건강, 질병이환, 건강수준, 건강행태 요인을 보정하여 성별로 층화하여 분석하였다. 저작 불편 호소 여부에 따른 우울증상은 남성의 경우 저작 불편을 호소하는 경우가 저작 불편을 호소하지 않는 경우에 비해 우울증상을 겪을 가능성이 1.45배(95% CI:1.29-1.63) 높았으며, 여성의 경우 1.50배(95% CI:1.39-1.61) 높았다. 발음 불편 호소 여부에 따른 우울증상은 남성의 경우 발음 불편을 호소하는 경우가 발음 불편을 호소하지 않는 경우에 비해 우울증상을 겪을 가능성이 1.97배(95% CI:1.76-2.20) 높았으며, 여성의 경우에는 1.55배(95% CI:1.44-1.67) 높았다.

연령의 경우 후기 노인이 전기 노인에 비해 우울증상을 겪을 가능성이 남성, 여성 모두에서 1.36배(95% CI:1.28-1.50) 높은 것으로 나타났으며, 교육수준의 경우 남성에서

고등학교 졸업이상인 경우에 비해 초등학교~중학교 졸업인 경우 우울증상을 겪을 가능성이 1.19배(95% CI:1.05-1.34), 여성에서 1.20배(95% CI:1.02-1.41) 높았다. 무학인 경우에 우울증상을 겪을 가능성이 남성에서 1.70배(95% CI:1.48-1.95) 여성에서 1.55배(95% CI:1.32-1.82) 높았다. 기초생활수급자가 아닌 경우에 비해 기초생활수급자인 경우 우울증상을 겪을 가능성이 남성에서 2.57배(95% CI:2.23-2.96), 여성에서 1.95배(95% CI:1.79-2.12)로 비교적 남성에서 높게 나타났다.

건강수준 요인을 살펴보면, 1~2개의 만성질환을 보유하고 있는 경우가 만성질환을 보유하지 않고 있는 경우에 비해 우울증상을 겪을 가능성은 남성에서 1.27배(95% CI:1.14-1.42), 여성에서 1.19배(95% CI:1.09-1.29) 높았고, 3개 이상의 복합만성질환을 보유하고 있는 경우 우울증상을 겪을 가능성은 남성에서 1.92배(95% CI:1.66-2.23), 여성에서 1.73배(95% CI:1.58-1.91) 높았다. 스트레스를 적게 느끼거나 거의 느끼지 않는 경우에 비해 스트레스를 많이 느끼는 경우 우울증상을 겪을 가능성은 남성에서 7.86배(95% CI:7.13-8.66), 여성에서 6.53배(95% CI:6.13-6.97) 높은 것으로 나타났다.

건강행태 요인을 살펴보면, 평생 5갑(100개비) 이상의 담배를 피우지 않은 경우에 비해 현재 흡연을 하고 있는 경우 우울증상을 겪을 확률은 남성에서 1.11배(95% CI:0.97-1.26), 여성에서 1.16배(95% CI:1.02-1.33) 높았으며, 과거에는 피웠으나 현재는 피우지 않는 경우 우울증상을 겪을 확률은 남성에서 0.94배(95% CI:0.83-1.06), 여성에서 1.37배(95% CI:1.16-1.61) 높았다. 평생 술을 마셔본 적이 없는 경우에 비해 최근 1년동안 금주한 경우 우울증상을 겪을 확률은 남성에서 1.31배(95% CI:1.14-1.50), 여성에서 1.18배(95% CI:1.08-1.29) 높았다. 하루 평균 수면시간의 경우 정상수면(6시간~8시간 이하) 습관을 가진 경우에 비해 수면 부족(6시간 미만)인 경우 우울증상을 겪을 확률은 남성에서 1.66배(95% CI:1.49-1.85), 여성에서 1.57배(95% CI:1.47-1.68) 높았으며, 낮잠시간을 제외하고도 하루 평균 수면시간이 8시간이상인 경우 우울증상을 겪을 확률은 남성에서 1.43배(95% CI:1.23-1.66), 여성에서 1.40배(95% CI:1.25-1.57) 높았다(Table 5).

고찰

노인 인구에서 저작기능과 같은 구강건강의 저하가 있는 경우 우울증 증상이 높은 것으로 보고되고 있는데[15], 본 연구에서도 이와 마찬가지로 저작 불편 및 발음 불편이 있을

수록 우울증상이 높게 나타났다. 인구사회학적 특성, 질병이환, 건강수준, 건강행태 요인을 모두 보정한 후에도 저작 불편을 호소하는 남성 노인의 경우 우울증상이 생길 가능성이 저작 불편을 호소하지 않는 남성 노인에 비해 1.45배(95% CI:1.29-1.63) 높았다. 이는 국민건강영양조사 제5기 1차년도(2010년) 자료를 이용한 연구에서 한국 성인의 저작불편감이 높을수록 생애 우울증을 겪을 가능성이 1.25배(95% CI:1.05-1.50) 높았다는 결과[16]와도 유사하였으며, 여성의 경우 저작불편을 호소하지 않는 여성에 비해 저작불편을 호소하는 여성이 우울증상을 겪을 가능성이 1.50배(95% CI:1.39-1.61)로 높게 나타나 저작불편을 호소하는 남성 노인에서보다 저작불편을 호소하는 여성노인에서 우울증상을 겪을 가능성이 더 높게 나타나 성별에 따른 차이가 있다는 것을 알 수 있었다.

발음 불편을 호소하는 남성 노인에서 우울증상을 겪을 가능성이 발음 불편을 호소하지 않는 남성 노인에 비해 1.97배(95% CI:1.76-2.20) 높았으며, 발음 불편을 호소하는 여성노인에서는 우울증상을 겪을 가능성이 1.55배(95% CI:1.44-1.67) 높아 임상적 구강질환이 정신건강의 요소인 우울에 강한 영향을 미친다는 보고[17]와도 유사한 결과로 나타났다. 중년기에는 사회적 활동이 많아지고 대화할 시간이 많아지면서 말하기에 문제가 있으면 우울증상을 겪는 것으로 보이는 것[18]과는 달리 노년기에는 경제활동시간이 줄어들면서 상대적으로 여가시간이 늘어나고, 식사 및 간식시간이 늘어나게 되면서 그동안 간과하였던 구강의 기능적인 상실에서 오는 절망감으로 인하여 우울증상을 겪는 것으로 보인다. 발음 불편을 호소하는 여성 노인에 비해 남성 노인에서 우울증상을 겪을 가능성이 높게 나타난 것은 남성이 여성보다 경제활동을 하는 시간이 상대적으로 길기 때문인 것으로 해석된다. 건강한 노년기를 보낼 수 있도록 지역사회에 거주하는 노인을 대상으로 구강의 기능적 회복을 통해 정신건강을 향상시키고, 구강건강 프로그램과 정신건강 프로그램을 중재하는 프로그램을 마련하는 등 다방면의 노력이 필요할 것으로 사료된다.

교육수준은 남성 노인에 비해 여성 노인에서 중학교 졸업 이하의 분율이 높게 나타났고, 기초생활수급자는 여성 수급자가 남성 수급자보다 더 많았다. 교육수준이 낮을수록 우울증상 유병률이 높게 나타났으며, 이는 기초생활수급자일수록, 교육수준이 낮

을수록 노인우울증의 유병율이 유의미하게 높아졌다는 연구결과와도 유사하였다[19]. 본 연구에서는 일반적인 사회경제적 수준만을 고려하였으나 향후 사회적 연결망, 지역적인 격차까지 고려한 연구도 필요할 것이라고 사료된다.

건강보험심사평가원에서 제공하는 2011년도의 환자표본자료를 활용한 자료에서 65세 이상 고령자의 만성질환 분포를 살펴보면, 만성질환이 없는 경우는 전체의 4.7%에 불과하였으며, 만성질환을 1개보유한 경우는 14.1%, 2개를 보유한 경우는 20.7%, 3개 이상을 보유한 경우는 60.5%였다[20]. 본 연구에서는 만성질환을 한 개 이상 보유하고 있는 노인이 만성질환을 하나도 보유하지 않은 노인에 비해 우울증상 유병률이 높게 나타났으며, 남성에서보다 여성에서 우울증상 유병률이 더 높게 나타났다. 만성질환을 3개 이상 보유한 복합만성질환자의 분율이 여성에서 높게 나타났으며, 2009년 자료를 분석하였다는 점을 고려해 볼 때, 두 개 또는 그 이상의 만성질환을 보유한 복합만성질환자는 앞으로 더욱 증가할 가능성이 높아 보인다. 우울증상을 겪고 있는 노인군에서 우울에 영향을 미치는 요인으로는 만성질환 개수, 통증, 인지기능을 들 수 있는데, 우울과 건강과의 관련성은 인과관계를 명확히 파악할 수 있는 요인들이라기보다는 우울이 건강에 영향을 미치고 반대로 건강이 우울에 영향을 미치는 복합적인 관계에 있다고 할 수 있다[21].

65세 이상 인구의 절반 이상은 일상생활에 스트레스를 거의 느끼지 않는 것으로 났으며, 여성이 남성보다 스트레스를 더 많이 느끼는 것으로 나타났는데, 이는 65세 이상에서 경제활동률이 낮다는 것을 고려해 볼 때 가정생활에 있어서 남성이 상대적으로 여성에 비해 스트레스를 덜 느끼는 것이라고 해석되어 진다. 스트레스가 많을수록 우울증상 유병률은 높은 것으로 나타났는데, 이는 스트레스(β=.53, p<.001)가 심할수록 우울로 가는 경로가 유의하였다는 선행연구[22]의 결과와 유사하였다. 기존의 노인은 스트레스 상황에서 쉽게 영향을 받으며 이러한 스트레스는 우울에 영향을 미치고 스트레스와 우울은 자살생각을 높게 할 수 있다[23]는 연구결과와 스트레스가 구강 점막 질환, 구강건조감에 영향을 미치고, 구강 점막 질환, 구강건조감은 스트레스 증상에 영향을 미친다[24]는 연구 결과 등을 종합해 볼 때 구강건강을 유지·증진시

키고, 스트레스를 낮춤으로써 우울증상을 낮출 수 있는 요인으로 볼 수 있다.

건강행태 요인을 살펴보면, 비경험자에 비해 흡연경험자(과거흡연, 현재흡연)의 경우 우울증상 유병률이 높게 나타났으며, 남성 흡연경험자보다 여성 흡연경험자에서 우울증상 유병률이 높게 나타났다. 건강이 악화되는 노년기에 들어서도 흡연을 지속하는 노인들은 노년기의 금연이 별 이득이 없다는 잘못된 인식이 높아 금연에 대한 동기가 부족하며 그 중 특히, 여성은 남성보다 우울 성향 및 스트레스 정도가 높아 담배에 대한 의존이 심해 남성보다 담배 끊기를 더 어려워하는 것으로 보인다[25]. 노인의 음주율은 남녀 모두 흡연율에 비해 높은 분율을 보였는데, 이와 같은 결과는 노인은 사랑하는 사람의 죽음, 은퇴, 만성질환, 불면증 등과 같은 위험요인에 노출되어 있기 때문인 것으로 생각해 볼 수 있다. 남성의 경우 음주경험자를 분율이 높게 나타나고 여성에서 우울이 더 심각하게 나타나는 양상을 보였다. 음주 경험자 중 과거 음주자에서 우울증 유병률이 높게 나타났는데, 이는 문제 음주자가 나이가 들면서 건강상의 이유 등으로 금주를 하게 됨으로써 우울증상을 겪게 되기 때문인 것으로 생각된다. 한편, 현재 음주자는 비음주자에 비해 우울증 유병률이 낮게 나타났는데 이는 우울할수록 음주량이 많았다는 연구결과[26]와 유사하였다. 하루 평균 수면시간에서 눈여겨 보아야 할 점은 수면과다와 수면부족인데 수면과다증과 불면증은 수면장애로 분류되므로 불면증 뿐 아니라 수면과다증에 대한 관심도 기울여야 할 필요가 있다. 본 연구에서 수면시간은 낮잠시간을 제외한 시간으로 낮잠시간이 많은 노인에서 낮잠시간을 제외한 수면시간이 8시간 이상이라면 수면과다증을 의심해 볼 수 있다. 우울증이 없는 사람에게도 불면증을 경험한 횟수가 많을수록 우울증이 발생할 확률이 2.5배 높았다는 결과[27]와 마찬가지로 불면증 또는 수면과다증과 같이 수면장애가 의심되는 그룹은 정상 수면시간을 유지하는 그룹에 비해 우울증상 유병률이 높게 나타났다.

본 연구결과와 선행연구 결과로 볼 때, 노인의 구강건강과 우울증상은 밀접하게 연관되어 나타나고, 우울증상 유병률이 계속 증가하는 추세라면 구강건강 관련 사업을 개발함에 있어 노인의 구강보건관리와 우울증관리를 중재하는 프로그램을 마련해야 하며, 체계적이고 지속가능한 프로그램을 통해 노인을 대상으로 맞춤형 보건교육을 실시하고, 정기

적인 치과내원을 통해 구강건강을 유지·증진시키려는 노력이 필요할 것으로 생각된다.

Table 1. General characteristics of study subjects by gender

		Male (n=20,582)		Female (n=30,112)	
		n	%	n	%
Age(years)	65-74	14,420	(70.1)	18,997	(63.1)
	≥75	6,162	(29.9)	11,115	(36.9)
Educational level	High school and over	5,482	(26.6)	1,555	(5.2)
	Elementary~Middle school	10,913	(53.0)	10,852	(36.0)
	None	4,187	(20.3)	17,705	(58.8)
National basic livelihood	No	19,238	(93.5)	26,676	(88.6)
security	Yes	1,344	(6.5)	3,436	(11.4)
No. of chronic diseases	0	7,345	(35.7)	5,539	(18.4)
	1~2	11,007	(53.5)	16,885	(56.1)
	≥3	2,230	(10.8)	7,688	(25.5)
Perceived stress	No	17,210	(83.6)	22,885	(76.0)
	Yes	3,372	(16.4)	7,227	(24.0)
Cigarette smoking	Never	5,125	(24.9)	27,759	(92.2)
	Past	9,399	(45.7)	893	(3.0)
	Current	6,058	(29.4)	1,460	(4.9)
Alcohol drinking	Never	4,102	(19.9)	19,391	(64.4)
	Past	5,132	(24.9)	4,006	(13.3)
	Current	11,348	(55.1)	6,715	(23.3)
Sleeping hours	6~8 hours less	14,904	(72.4)	20,219	(67.2)
	<6 hours	3,794	(18.4)	7,650	(25.4)
	≥8 hours	1,884	(9.2)	2,243	(7.5)
Chewing discomfort	No	10,510	(51.1)	13,464	(44.7)
	Yes	10,072	(48.9)	16,648	(55.3)
Pronouncing discomfort	No	15,304	(74.4)	21,633	(71.8)
	Yes	5,278	(25.6)	8,479	(28.2)
Depressive symptoms (CES-D)	No(<16)	17,966	(87.3)	23,097	(76.7)
	Yes(≥16)	20,582	(12.7)	7,015	(23.3)

Table 2. Distribution of factors related to Chewing discomfort by gender

	Male (n= 20,582)					Female (n= 30,112)				
	No. Sub♯	Weighed prevalence		Age adjusted prevalence*		No. Sub♯	Weighed prevalence		Age adjusted prevalence*	
		%	SE	%	(95% CI)		%	SE	%	(95% CI)
Educational level										
High school and over	5,482	35.8	(0.6)	34.6	(32.8,36.4)	1,555	32	(1.2)	34	(30.9,37.1)
Elementary~Middle school	10,913	50.7	(0.5)	48.9	(47.5,50.3)	10,852	48	(0.5)	48.3	(46.9,49.7)
None	4,187	61.6	(0.8)	57.8	(55.4,60.2)	17,705	61.8	(0.4)	58.5	(57.3,59.7)
National basic livelihood security										
No	19,238	47.8	(0.4)	44.2	(43.2,45.2)	26,676	53.4	(0.3)	50.5	(49.7,51.3)
Yes	1,344	65.8	(1.3)	61.5	(57.6,65.4)	3,436	69.8	(0.8)	65.9	(63.5,68.3)
No. of chronic diseases										
0	7,345	47.1	(0.6)	43.5	(41.9,45.1)	5,539	49.8	(0.7)	46.8	(44.8,48.8)
1~2	11,007	48.9	(0.5)	44.6	(43.2,46.0)	16,885	54.5	(0.4)	50.5	(49.3,51.7)
≥3	2,230	55.2	(1.1)	52.3	(49.4,55.2)	7,688	61.1	(0.6)	58	(56.4,59.6)
Perceived stress										
No	17,210	46.1	(0.4)	42	(41.0,43.0)	22,885	51.5	(0.3)	47.4	(46.4,48.4)
Yes	3,372	63.4	(0.8)	60.4	(58.0,62.8)	7,227	67.2	(0.6)	65.2	(63.6,66.8)
Cigarette smoking										
Never	5,125	40.4	(0.7)	36.5	(34.7,38.3)	27,759	54.2	(0.3)	51.2	(50.4,52.0)
Past	9,399	49.5	(0.5)	44.3	(42.9,45.7)	893	69.8	(1.5)	65.6	(60.5,70.7)
Current	6,058	55.3	(0.6)	54.2	(52.4,56.0)	1,460	67.4	(1.2)	61.8	(57.9,65.7)
Alcohol drinking										
Never	4,102	49.2	(0.8)	43.8	(41.6,46.0)	19,391	54.9	(0.4)	51.1	(50.1,52.1)
Past	5,132	54.9	(0.7)	50.8	(48.8,52.8)	4,006	61.3	(0.8)	58.2	(56.0,60.4)
Current	11,348	46.2	(0.5)	43.5	(42.1,44.9)	6,715	52.9	(0.6)	50.5	(48.7,52.3)
Sleeping hours										
6~8 hours less	14,904	46.6	(0.4)	43.1	(41.9,44.3)	10,638	52.6	(0.4)	49.6	(48.6,50.6)
<6 hours	3,794	54.9	(0.8)	50.7	(48.3,53.1)	4,684	61.2	(0.6)	57.3	(55.7,58.9)
≥8 hours	1,844	55.1	(1.1)	51.4	(47.9,54.9)	1,326	59.1	(1.0)	55.3	(52.0,58.6)

♯No. Sub, number of subject, ¶ No. Chew, number of chewing discomfort
* Adjusted for age by direct standardization using 2005 Census as standard population
SE, standard error.

Table 3. Distribution of factors related to Pronouncing discomfort by gender

	Male (n= 20,582)					Female (n= 30,112)				
	No. Sub♯	Weighed prevalence		Age adjusted prevalence*		No. Sub♯	Weighed prevalence		Age adjusted prevalence*	
		%	SE	%	(95% CI)		%	SE	%	(95% CI)
Educational level										
High school and over	5,482	17	(0.5)	15.8	(14.6,17.0)	1,555	13.7	(0.9)	14.4	(12.0,16.8)
Elementary~Middle school	10,913	25.9	(0.4)	24.4	(23.2,25.6)	10,852	21.9	(0.4)	22	(20.8,23.2)
None	4,187	36.3	(0.7)	32.2	(29.8,34.6)	17,705	33.2	(0.4)	30.3	(29.3,31.3)
National basic livelihood security										
No	19,238	24.5	(0.3)	21.7	(20.9,22.5)	26,676	26.6	(0.3)	24.2	(23.4,25.0)
Yes	1,344	41.5	(1.3)	37.8	(34.1,41.5)	3,436	40.6	(0.8)	37.8	(35.4,40.2)
No. of chronic diseases										
0	7,345	24	(0.5)	20.8	(19.4,22.2)	5,539	27.1	(0.6)	24	(22.4,25.6)
1~2	11,007	25.8	(0.4)	22.5	(21.5,23.5)	16,885	27.4	(0.3)	24.3	(23.3,25.3)
≥3	2,230	30.5	(1.0)	27.6	(25.1,30.1)	7,688	30.5	(0.5)	28.8	(27.4,30.2)
Perceived stress										
No	17,210	23.4	(0.3)	20.1	(19.3,20.9)	22,885	26	(0.3)	22.9	(22.1,23.7)
Yes	3,372	36.9	(0.8)	34.7	(32.5,36.9)	7,227	35.1	(0.6)	33.4	(31.8,35.0)
Cigarette smoking										
Never	5,125	23.1	(0.6)	19.2	(17.8,20.6)	27,759	27.3	(0.3)	24.8	(24.0,25.6)
Past	9,399	25.3	(0.4)	21.7	(20.5,22.9)	893	37	(1.6)	33.6	(28.9,38.3)
Current	6,058	28.3	(0.6)	27	(25.4,28.6)	1,460	39.2	(1.3)	36.5	(33.0,40.0)
Alcohol drinking										
Never	4,102	28.8	(0.7)	24.6	(22.8,26.4)	19,391	29.1	(0.3)	26	(25.2,26.8)
Past	5,132	32.3	(0.7)	28.6	(26.8,30.4)	4,006	31.1	(0.7)	29.3	(27.3,31.3)
Current	11,348	21.5	(0.4)	19.5	(18.5,20.5)	6,715	23.6	(0.5)	21.5	(20.1,22.9)
Sleeping hours										
6~8 hours less	14,904	23.8	(0.3)	20.8	(19.8,21.8)	20,219	26.7	(0.3)	24.2	(23.4,25.0)
<6 hours	3,794	29.5	(0.7)	26.5	(24.5,28.5)	7,650	30.3	(0.5)	27.8	(26.4,29.2)
≥8 hours	1,884	32.3	(1.1)	28.5	(25.6,31.4)	2,243	34.2	(1.0)	28.4	(25.9,30.9)

♯No. Sub, number of subject, ¶ No. Chew, number of chewing discomfort
* Adjusted for age by direct standardization using 2005 Census as standard population
SE, standard error.

Table 4. Distribution of factors related to Depressive symptoms by gender

	Male (n= 20,582)					Female (n= 30,112)				
	No. Sub♯	Weighed prevalence		Age adjusted prevalence*		No. Sub♯	Weighed prevalence		Age adjusted prevalence*	
		%	SE	%	(95% CI)		%	SE	%	(95% CI)
Educational level										
High school and over	5,482	9.3	(0.4)	10.1	(8.9,11.3)	1,555	14.6	(0.9)	16.2	(13.7,18.7)
Elementary~Middle school	10,913	12	(0.3)	13.3	(12.3,14.3)	10,852	19.2	(0.4)	21.5	(20.3,22.7)
None	4,187	18.9	(0.6)	20.6	(18.4,22.8)	17,705	26.6	(0.3)	29.6	(28.6,30.6)
National basic livelihood security										
No	19,238	11.3	(0.2)	11.8	(11.2,12.4)	26,676	20.8	(0.2)	22.4	(21.5,23.1)
Yes	1,344	32.6	(1.3)	35.5	(31.8,39.2)	3,436	42.6	(0.8)	48.1	(45.6,50.6)
No. of chronic diseases										
0	7,345	9.7	(0.3)	10.2	(9.2,11.2)	5,539	16.9	(0.5)	17.8	(16.2,19.4)
1~2	11,007	13	(0.3)	13.5	(12.5,14.5)	16,885	21.4	(0.3)	22.6	(21.4,23.8)
≥3	2,230	21.2	(0.9)	19.4	(17.2,21.6)	7,688	32.1	(0.5)	33.7	(32.1,35.3)
Perceived stress										
No	17,210	7.2	(0.2)	7.4	(6.8,8.0)	22,885	13.7	(0.2)	14.6	(13.8,15.4)
Yes	3,372	41	(0.8)	41.5	(39.1,43.9)	7,227	53.8	(0.6)	55.4	(53.8,57.0)
Cigarette smoking										
Never	5,125	11	(0.4)	10.7	(9.5,11.9)	27,759	22.5	(0.3)	24.1	(23.3,24.9)
Past	9,399	12.7	(0.3)	13	(12.0,14.0)	893	35.3	(1.6)	37.5	(32.6,42.4)
Current	6,058	14.3	(0.4)	15.7	(14.3,17.1)	1,460	31.8	(1.2)	36.4	(32.7,40.1)
Alcohol drinking										
Never	4,102	12.8	(0.5)	13.2	(11.6,14.8)	19,391	23.2	(0.3)	24.4	(23.4,25.4)
Past	5,132	18.9	(0.5)	19.4	(17.8,21.0)	4,006	29.6	(0.7)	31.5	(29.5,33.5)
Current	11,348	9.9	(0.3)	10.5	(9.7,11.3)	6,715	20	(0.5)	22.4	(20.8,24.0)
Sleeping hours										
6~8 hours less	14,904	10.3	(0.2)	10.8	(10.0,11.6)	20,219	19	(0.3)	20.8	(20.0,21.6)
<6 hours	3,794	20.3	(0.7)	20.7	(18.9,22.5)	7,650	33.8	(0.5)	34.6	(33.0,36.2)
≥8 hours	1,884	16.2	(0.8)	16.9	(14.2,19.6)	2,243	26.2	(0.9)	26.7	(24.0,29.4)
Chewing discomfort										
No	10,510	7.1	(0.3)	7.6	(6.8,8.4)	13,464	14.6	(0.3)	16.1	(15.1,17.1)
Yes	10,072	18.5	(0.4)	19.9	(18.7,21.1)	16,648	30.3	(0.4)	33.3	(32.3,34.3)
Pronouncing discomfort										
No	15,304	8.8	(0.2)	9.2	(8.6,9.8)	21,633	18.8	(0.3)	20.5	(19.7,21.3)
Yes	5,278	24.2	(0.6)	26.8	(24.8,28.8)	8,479	34.9	(0.5)	38.3	(36.7,39.9)

♯No. Sub, number of subject, ¶ No. Chew, number of chewing discomfort
* Adjusted for age by direct standardization using 2005 Census as standard population
SE, standard error.

Table 5. The relationship between oral health and depressive symptoms in elderly(odds ratios and 95% confidence intervals)

		Male (n=20,582)								Female (n=30,112)							
		model 1		model 2		model 3		model 4		model 1		model 2		model 3		model 4	
		OR	(95%, CI)	OR	(95%, CI)	OR	(95%, CI)	OR	(95%, CI)	OR	(95%, CI)	OR	(95%, CI)	OR	(95%, CI)	OR	(95%, CI)
Chewing discomfort	No	1	(referent)	1	(referent)	1	(referent)	1	(referent)	1	(referent)	1	(referent)	1	(referent)	1	(referent)
	Yes	1.93	(1.73-2.15)	1.78	(1.60-1.99)	1.48	(1.32-1.66)	1.45	(1.29-1.63)	2.02	(1.89-2.16)	1.87	(1.75-2.00)	1.54	(1.43-1.65)	1.50	(1.39-1.61)
	p-value		<0.0001		<0.0001		<0.0001		<0.0001		<0.0001		<0.0001		<0.0001		<0.0001
Pronouncing discomfort	No	1	(referent)	1	(referent)	1	(referent)	1	(referent)	1	(referent)	1	(referent)	1	(referent)	1	(referent)
	Yes	2.3	(2.08-2.54)	2.12	(1.91-2.35)	2.04	(1.83-2.28)	1.97	(1.76-2.20)	1.62	(1.51-1.72)	1.52	(1.42-1.62)	1.55	(1.45-1.67)	1.55	(1.44-1.67)
	p-value		<0.0001		<0.0001		<0.0001		<0.0001		<0.0001		<0.0001		<0.0001		<0.0001
Age(years)	65-74			1	(referent)	1	(referent)	1	(referent)			1	(referent)	1	(referent)	1	(referent)
	≥75			1.22	(1.11-1.33)	1.44	(1.30-1.59)	1.36	(1.28-1.50)			1.08	(1.02-1.15)	1.42	(1.33-1.51)	1.36	(1.28-1.45)
	p-value				<0.0001		<0.0001		<0.0001				0.0084		<0.0001		<0.0001
Educational level	High school and over			1	(referent)	1	(referent)	1	(referent)			1	(referent)	1	(referent)	1	(referent)
	Elementary~Middle school			1.09	(0.98-1.22)	1.2	(1.07-1.36)	1.19	(1.05-1.34)			1.19	(1.02-1.39)	1.19	(1.01-1.40)	1.2	(1.02-1.41)
	p-value				0.0091		0.0442		0.0546				0.5555		0.3495		0.4628
	None			1.5	(1.32-1.70)	1.75	(1.52-2.01)	1.7	(1.48-1.95)			1.49	(1.29-1.73)	1.55	(1.32-1.82)	1.55	(1.32-1.82)
	p-value				<0.0001		<0.0001		<0.0001				<0.0001		<0.0001		<0.0001
National basic livelihood security	No			1	(referent)	1	(referent)	1	(referent)			1	(referent)	1	(referent)	1	(referent)
	Yes			3.01	(2.65-3.42)	2.72	(2.36-3.13)	2.57	(2.23-2.96)			2.41	(2.23-2.60)	1.02	(1.86-2.20)	1.95	(1.79-2.12)
	p-value				<0.0001		<0.0001		<0.0001				<0.0001		<0.0001		<0.0001
No. of chronic diseases	0					1	(referent)	1	(referent)					1	(referent)	1	(referent)
	1~2					1.29	(1.16-1.43)	1.27	(1.14-1.42)					1.19	(1.09 -1.30)	1.19	(1.09-1.29)
	p-value						0.0532		0.0793						0.0003		0.0011
	≥3					2	(1.73-2.31)	1.92	(1.66-2.23)					1.78	(1.62-1.96)	1.73	(1.58-1.91)
	p-value						<0.0001		<0.0001						<0.0001		<0.0001
Perceived stress	No					1	(referent)	1	(referent)					1	(referent)	1	(referent)
	Yes					1.29	(1.16-1.43)	7.86	(7.13-8.66)					6.89	(6.47-7.34)	6.53	(6.13-6.97)
	p-value						<0.0001		<0.0001						<0.0001		<0.0001
Cigarette smoking	Never							1	(referent)							1	(referent)
	Past							0.94	(0.83-1.06)							1.37	(1.16-1.61)
	p-value								0.0199								0.0063
	Current							1.11	(0.97-1.26)							1.16	(1.02-1.33)
	p-value								0.0111								0.9167
Alcohol drinking	Never							1	(referent)							1	(referent)
	Past							1.31	(1.14-1.50)							1.18	(1.08-1.29)
	p-value								<0.0001								<0.0001
	Current							0.78	(0.69-0.89)							0.86	(0.79-0.93)
	p-value								<0.0001								<0.0001
Sleeping hours	6~8 hours less							1	(referent)							1	(referent)
	<6 hours							1.66	(1.49-1.85)							1.57	(1.47-1.68)
	p-value								<0.0001								<0.0001
	≥8 hours							1.43	(1.23-1.66)							1.4	(1.25-1.57)
	p-value								0.1818								0.0443

References

1. Suh UJ, Lee JY, Estimated future population(2010~2060);2010[cited 2011 Dec 7]. Available from:http://meta.narastat.kr/metasvc/index.do?confmNo=10133&inputYear=2011/estimated future population 2010 2060.pdf.

2. Han DH. Chewing difficulty and multiple chronic conditions in Korean elders: KNHANES IV. The Journal of Koeran Dental Association 2013;51:511-517.

3. Health Insurance Review & Assessment Service:2014[cited 2015 Feb]. Available from: http://www.hira.or.kr/dummy.do?pgmid=HIRAA020041000000&cmsurl=/cms/notice/02/1332409_24959.html&subject#none/20150225_2014.pdf.

4. Lee HL, Lee HM, Kim HJ, Oh KW. Trends Oral Health Status among Adults Over 65 Years Old in Korea, 2007-2013. Public health weekly report, KCDC 2015;8:735-736.

5. Kim SK, Kwon MY. Analysis of satisfaction level regarding removable dentures for aged patients in dental hospitals or clinics. J Kor Soc Dent Hygiene 2006;6:157-168.

6. Oh JK, Kim YJ, Kho HS. A Study on the Clinical Characteristics of Patients with Dry Mouth. Korean Journal of Oral Medicine 2001;26:331-343.

7. Kim YA. Depression Symptoms Experience Among Adults in Korea, 2012. Public health weekly report, KCDC 2014;7:819-820.

8. Lee HS, Kim CM. Effects of Oral Health Impact Profile (OHIP) on Depression and Quality of Life among Community-dwelling Korean Elderly Persons. J Kor Acad Community Health Nurs 2012;23:338-346.

9. Kim KW. The Effects of Oral Exercise on Xerostomia and Depression Scale among the Elderly Patients. The Journal of The Korean Academy of Dental Hygiene 2014; 16:61-69.

10. Bojorquez-Chapela I, Villalobos-Daniel VE, Manrique-Espinoza BS, Tellez-Rojo MM, Salinas-Rodríguez A. Depressive symptoms among poor older adults in Mexico: prevalence and associated factors. Rev Panam Salud Publica 2009;26:70-77.

11. Takiguchi T, Yoshihara A, Takano N, Miyazaki H. Oral health and depression in older

Japanese people. Gerodontology 2015;3.

12. Lee,HS, Kim CM. Effects of Oral Health Impact Profile(OHIP) on Depression and Quality of Life among Community-dwelling Korean Elderly Persons. J Korean Acad Community Health Nurs 2012;23:338-346.

13. Kim KW. The Effects of Oral Exercise on Xerostomia and Depression Scale among the Elderly Patients. J Korean Society of Dental Hygiene 2014;16:61-69.

14. Cho MJ, Kim KH. Diagnostic Validity of the CES-D(Korean Version) in the Assessment of DSM-III-R Major Depressiion. J Korean Neuropsychiatr Assoc, 1993;32:381-399.

15. Lee MA. Relative Effects of Health and Family Factors on Geriatric Depression. Kor.J Community Living Sci 2011;22:623-635.

16. Park SJ, Ko KD, Shin SI, Ha YJ, Kim GY, Kim HA. Association of oral health behaviors and status with depression: results from the Korean National Health and Nutrition Examination Survey, 2010. J Public Health Dent 2014;74:127-38.

17. Jang JH, Kin SH. The Relationship between Xerostomia and Depression in Elderly People. Journal of Korean Society for Health Education and Promotion 2007;24:51-60.

18. Lee HJ, Kahng SK, Lee JY. The Effects of Socioeconomic Position and Health Behavior on Geriatric Depressive Symptom. Journal of the Korean Gerontological Society 2008;28:1129-1145.

19. Jung YH, Koh SJ, Kim EJ. A study on the effective chronic disease management. Seoul:Korea Institute for Health and Social Affairs; 2013, p.1-152(Korean).

20. Jung YM. Health Status and Associated Factors of Depression according to Depression in Elderly Women. Journal of the Korean Gerontological Society 2007;27:71-86.

21. Woo HB. Differences in Healthy Life Expectancy by Gender and Education for

Middle-Aged and Older Korans. Korean Journal of Sociology 2009;43:165-187.

22. Jang MH, Kim YH. The Relationship of Stress, Depression and Suicidal Ideation in the Elderly, J Korean Acad Psych Mental Health Nurs 2005;14:33-42.

23. Hong MH. The influence of stress on oral mucosal disease, dry mouth and stress symptoms in adults. Journal of Korean Society of Dental Hygiene 2013;13:589-596.

24. Kim JS, Yu JO, Kim MS. Factors Contributing to the Intention to Quit Smoking in Community-dwelling Elderly Smokers. J Korean Acad Community Health Nurs 2012;23:358-365.

25. Stress, Depression and Health Risk Behaviors in Korean Elders. Korean Academy of Community Health Nursing 2007;18:391-399.

26. Kim AJ, Kim OS, Baik SH, Jang JH, Kim Jh. Suh SY, Kim H, Ynag HJ, Cho ER, Lee SK, Shin C. Longitudinal course of depression with and without insomnia in non-depressed individuals: A 6-year follow-up study. SLEEP 2013;36:369-376.

27. Park KH, Kim HS, Yi YJ. Comparison of Risk Factors in Cognitive Impairment between Non-depressive Elderly and Depressive Elderly. Journal of the Korea contents association 2013;13:301-31.
